# Impact of ABO blood type on the risk of atrial fibrillation recurrence after catheter ablation

**DOI:** 10.1016/j.ahjo.2024.100384

**Published:** 2024-03-15

**Authors:** Michitaka Amioka, Hiroki Kinoshita, Akinori Sairaku, Tomoki Shokawa, Yukiko Nakano

**Affiliations:** a Deparment of Cardiovascular Medicine, Hiroshima General Hospital, Hiroshima, Japan; b Deparment of Cardiovascular Medicine, Onomichi General Hospital, Hiroshima, Japan; c Deparment of Cardiovascular Medicine, NHO Higashihiroshima Medical Center, Hiroshima, Japan; d Deparment of Cardiovascular Medicine, Hiroshima University Graduate School of Biomedical and Health Sciences, Hiroshima, Japan

**Keywords:** ABO blood types, B antigen, Atrial fibrillation

## Abstract

**Background:**

Blood types are classified based on the specific antigenic characteristics they possess. Despite documented associations between antigens and inflammation, a scarcity of data exists concerning the impact of antigens on atrial fibrillation (AF).

**Methods:**

OSHOH-rhythm study is a multi-center, prospective observational study of 601 patients who underwent catheter ablation for AF. We examined the correlation between blood type groups and both the incidence and recurrence of AF. Additionally, we analyzed the recurrence of AF across antigenic profiles.

**Results:**

The frequencies of individual blood types were 239 (39.8 %), 190 (31.6 %), 122 (20.3 %), and 50 (8.3 %) for A, O, B, and AB, respectively, aligning closely with the prevalent blood type distribution among the Japanese populace. During follow-up period (18.8 months, median), AF recurrence occurred in 96 patients (22.4 %) lacking the B antigen (A and O), and 26 patients (15.1 %) possessing B antigen (B and AB), respectively (Log-rank test: *P* = 0.034). A multivariate analysis demonstrated that blood types lacking the B antigen (hazard ratio [HR], 1.55; 95 % CI, 1.01 to 2.42; *P* = 0.037), hypertension (HR, 1.51; 95 % CI, 1.05 to 2.17; *P* = 0.026) and non-paroxysmal AF (HR, 1.70; 95 % CI, 1.17 to 2.47; *P* = 0.005) were independently associated with the recurrence of AF.

**Conclusions:**

This study elucidates that, despite the absence of direct correlation between blood types and the occurrence of AF, blood types devoid of the B antigen exhibit an enhanced predisposition to AF recurrence. Nonetheless, the intricate mechanism linking blood type to recurrence remains elusive, warranting further comprehensive foundational research on blood types.

## Highlights

• The distribution of atrial fibrillation patients according to blood type was in concordance with the proportions of blood types observed within the Japanese populace.
• Notably, there was no clear difference in incidence of atrial fibrillation between the different blood types.
• Non-B antigen blood types (A and O) emerged as independent risk factors for the recurrence of atrial fibrillation after catheter ablation.
• This study constituted a research endeavor that elucidated variations in the recurrence rates of atrial fibrillation across different blood groups, thereby implying a novel clinical approach.

## 1 Introduction

Atrial fibrillation (AF) constitutes a prevalent cardiac ailment. While catheter ablation stands as an increasingly utilized therapeutic avenue for AF, a considerable proportion of patients experience recurrences. An investigation of the predictors of AF recurrence is difficult because the patient population in which AF occurs is heterogeneous and whether to predict a recurrence depends on the patient factors as well as technical aspects of the procedure [1,2]. Patient-specific variables, such as hypertension, diabetes, obesity, sleep apnea, alcohol consumption, sinus node dysfunction, and genetic factors, have been documented as factors associated with AF recurrence. Pulmonary vein (PV) reconnection, a technical facet of ablation, significantly influences the recurrence of AF [2]. According to the results of a prior meta-analysis, PV reconnection occurred in no fewer than one pulmonary vein in over 80 % of patients experiencing recurrence following the initial PV isolation [3]. However, technological advancements in catheter technology alone are inadequate to overcome this issue. Fibrotic and inflammatory biomarkers exhibit robust associations with the pathophysiology of AF and possess substantial predictive efficacy for AF recurrence [[4], [5], [6], [7], [8], [9]]. Given that cardiac inflammation underlies AF pathogenesis by provoking atrial electrical and structural remodeling, patients with heightened cardiac inflammation manifest augmented vulnerability or recurrence of AF post-catheter ablation [10]. Previous studies have elucidated a connection between ABO blood type and inflammation, thromboembolism, age-related diseases, and cardiovascular disease [11]. It is well known that patients with non-O blood types (A, B, or AB) have a higher risk of thromboembolic events, such as coronary artery disease and cerebrovascular ischemic events, and peripheral vascular disease, in stark contrast to their type O counterparts [12]. Liu et al. have reported that type O blood emerged as an independent predictor of very late recurrence subsequent to catheter ablation in patients with non-paroxysmal AF [13]. In accordance with the sugar chain composition, type A blood possesses *N*-acetylglucosaminyltransferase, responsible for A antigen production, while type B blood possesses galactosyltransferase, the generator of the B antigen. Type AB encompasses both, while type O features neither [14]. These transferases have been implicated in inflammatory processes and tissue healing. In this investigation, we scrutinized the occurrence and recurrence of AF in correlation with each blood type harboring antigens.

## 2 Methods

### 2.1 Study design

OSHOH-rhythm (Optimal Surveillance for protecting Heart disease in Onomichi and Hiroshima) study is a multi-center, non-randomized, open-labeled, prospective registry designed to assess the correlation between blood type groups and the incidence as well as recurrence of AF subsequent to catheter ablation. The institutional Ethics Board of Hiroshima General Hospital approved the study protocol and informed consent was obtained from all the patients before enrolment.

### 2.2 Patient selection and medications

The consecutive patients undergoing first AF ablation treatment at Hiroshima General Hospital, Onomichi General Hospital, and Hiroshima University Hospital between January 2017 and March 2022 were included. AF included paroxysmal, persistent, and permanent atrial fibrillation, which was either previously known or diagnosed during an outpatient visit. Patients below the age of 18, those lacking follow-up within the first year, individuals who succumbed during the follow-up period from any cause, those afflicted with heart failure due to advanced valvular disease, or those suffering from advanced dementia, were excluded from the analysis.

### 2.3 Electrophysiological study and RFCA

The patients were treated with warfarin or direct anticoagulants at least 1 month before the radiofrequency catheter ablation (RFCA) and throughout the periprocedural period. All antiarrhythmic drugs (AADs) other than amiodarone were stopped at least five half-lives before the RFCA. Amiodarone was discontinued at least 2 weeks before the RFCA. Mapping catheters (Lasso and/or PENTARAY; Biosense Webster, CA, USA) were positioned within the ipsilateral superior and inferior left pulmonary veins (PVs) under the guidance of selective PV angiography. A 6-F multipolar (15-pole) catheter (BeeAT, Japan Lifeline, Japan) was utilized with the distal poles (poles 1–8) placed within the coronary sinus and proximal electrodes (poles 9–15) located from the superior vena cava to the superior right atrium via the right internal jugular vein. A fixed multipolar electrode catheter (Abott, Chicago, USA) was introduced via the femoral vein to the His bundle and RV regions. Intravenous isoproterenol (ISP) was administered to the right femoral vein (10 μg flash) to induce AF before an extensive encircling pulmonary vein isolation (EEPVI). If AF was not induced, intravenous adenosine 5′-triphosphate (ATP) (10 mg) was administered immediately after the ISP infusion (10 μg flash). ISP was added if AF was not induced up to 30 μg. A continuous EEPVI was performed 0.5 to 2.0 cm from the PV ostia using a guided three-dimensional electroanatomical mapping system (CARTO® 3; Biosense Webster) with computed tomography integration (CARTO Merge; Biosense Webster) to achieve electrical isolation of the left and right PVs. An irrigated 3.5 mm tip electrode catheter (THERMOCOOL SmartTouch SF; Biosense Webster) was used for the EEPVI. A point-by-point ablation was performed. Radiofrequency energy was delivered at 35–40 W power with temperature limited to 45 °C. Successful PV isolation was defined as the loss of all PV potentials (entrance block) and failure to capture the left atrium when pacing from the PV (output 5 mA, pulse width 2 ms, exit block) using a circular multipolar mapping catheter under an injection of both ISP and ATP. Esophageal temperature monitoring was performed in all patients. No patient underwent extensive complex fractionated atrial electrogram ablation. Only patients with clinical or induced right atrial isthmus flutter underwent cavotricuspid isthmus (CTI) ablation. All non-CTI flutters and other atrial tachycardias (AT) were mapped and ablated using activation and entrainment mapping, if induced easily. Additionally, mitral isthmus ablation and/or roof-line ablation were rarely performed for the treatment of left atrium (LA) flutter and/or roof-dependent AT. Ablation in the superior vena cava (SVC) isolation were also rarely performed. Biphasic direct-current cardioversion (defibrillation) restored sinus rhythm if AF did not terminate spontaneously after successful PVI. The endpoint of the PVI was the creation of a bidirectional conduction block between the LA and the PVs. All PVs were successfully isolated following the procedure.

### 2.4 Follow-up after RFCA

After undergoing RFCA, patients were administered necessary medications. AADs, encompassing classes IA, IC, II, and III, as well as bepridil following the Vaughan–Williams classification, were employed to sustain sinus rhythm during the blanking period, either upon early recurrence of AF or when deemed essential by the attending physician. Patients were scheduled for regular assessments at 1, 3, and 6 months, followed by semiannual visits thereafter, in the outpatient clinic. At each clinical encounter, twelve‑lead electrocardiography, echocardiography, and 24-hour or one-week Holter monitoring were performed to ascertain the presence or absence of AF recurrence. Patients were explicitly instructed to seek medical attention in the outpatient clinic or emergency department in the event of palpitations or an irregular pulse. Upon detection of any abnormality, 24-hour Holter monitoring or a portable electrocardiography monitor was employed to confirm the presence of AF. AF recurrence was defined as an episode of palpitations lasting >30 s or as AF, atrial flutter, or atrial tachycardia episodes lasting >30 s, observed >3 months post-ablation.

### 2.5 Blood types

Blood typing was checked during the outpatient visit prior to catheter ablation. Standard slide method was adopted: a drop of each of the monoclonal antisera (Anti-A, Anti-B and Anti-D) was taken on glass slides. The blood cells subject of whose blood group is to be determined were mixed with each blood separately with the help of separate glass rods. Blood groups were determined on the basis of agglutination reaction within 5 min of mixing.

### 2.6 Clinical outcomes

Initially, we assessed the incidence and recurrence rates of AF stratified by blood types. The primary outcome centered on discerning variations in AF recurrence rates across distinct blood types. The outcome was primarily analyzed as the time to the first event. Subsequently, based on the results, we conducted a comparative analysis of the disparities in recurrence rates among different antigens. Secondary outcomes were dedicated to elucidating distinctions in recurrence rates across the antigens.

### 2.7 Statistical analysis

The continuous variables are summarized as the means ± standard deviation (SD). Categorical variables are expressed as absolute numbers and percentages. The differences in the categorical variables across the each of blood types were examined with the use of a Chi-Square test or Fisher's exact test. A Student's one-way analysis of variance was used to assess inter-group differences in normally distributed variables, whereas Tukey's post hoc test was used to determine inter-group statistical significance. A Kaplan-Meier analysis was used to assess the time required for the primary composite outcome. A Wilcoxon log-rank test was applied to compare the differences between the groups for the incidence of each of the primary outcomes. Univariate and multivariate Cox proportional hazard models were used to determine the predictors of the primary outcome after adjustments for potential confounding factors and with the use of stepwise methods. *P* values <0.05 were considered statistically significant. All statistical analyses were performed with the use of JMP software version 17 (SAS Institute, Cary, NC, USA).

## 3 Results

### 3.1 Patient characteristics

**Fig. 1:**
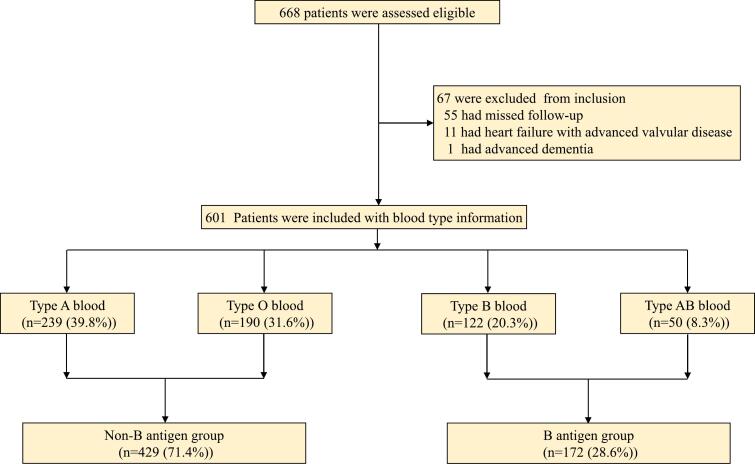
A flow chart detailing the study design. After the exclusion of 67 patients with any reasons, a total of 601 patients who were enrolled with blood type information was available.

During the study period, 55 patients withdrew from the study for various reasons. Eleven patients developed heart failure attributable to progressive valvular disease and were consequently excluded. One patient was withdrawn due to advanced dementia. A total of 67 patients met the exclusion criteria, and therefore consecutive 601 patients meeting the inclusion criteria were registered (Fig. 1). Among these, the distribution of A, O, B, and AB blood types was 239 (39.8 %), 190 (31.6 %), 122 (20.3 %), and 50 (8.3 %), respectively. A comprehensive summary of the demographics and baseline data for each blood type group is presented in Table 1. The median duration of follow-up for the primary outcome was 18.8 months (standard deviation, 9.2 months). The mean age was 66.8 years (standard deviation, 10.5 years), with 413 patients (68.7 %) being male. Pharmacological interventions post-blanking period relative to blood types were shown in Table 2. Subsequent to catheter ablation, beta-blockers and antiarrhythmic medications were sustained in 40.1 % and 16 % of patients, respectively. Additionally, eighteen patients were prescribed two or more antiarrhythmic drugs. Notably, there were no significant differences observed in baseline characteristics among the various blood types.

**Table 1:** Baseline characteristics between the blood type.

Blood type	All	A	O	B	AB	P value
N, (%)	601 (100 %)	239 (39.8 %)	190 (31.6 %)	122 (20.3 %)	50 (8.3 %)	
Variable						
Age, years	66.9 ± 10.6	66.9 ± 10.3	66.9 ± 10.9	66.4 ± 10.2	67.4 ± 9.7	0.973
Male, n (%)	413 (68.7 %)	160 (66.9 %)	136 (71.6 %)	88 (72.1 %)	29 (58 %)	0.119
BMI, kg/m^2^	24.2 ± 3.8	24.4 ± 4.1	24.2 ± 3.5	24.2 ± 3.7	23.4 ± 3.1	0.375
Medical history						
Hypertension, n (%)	357 (59.4 %)	148 (61.9 %)	110 (57.9 %)	69 (56.6 %)	30 (60 %)	0.893
Diabetes mellitus, n (%)	125 (20.8 %)	51 (21.3 %)	39 (20.5 %)	19 (15.6 %)	16 (32 %)	0.128
Heart failure etiology, n (%)	63 (10.5 %)	25 (10.5 %)	18 (9.5 %)	16 (13.1 %)	4 (9.5 %)	0.683
Paroxysmal atrial fibrillation, n (%)	427 (71 %)	171 (71.5 %)	132 (69.5 %)	89 (73 %)	35 (70 %)	0.921
CHADS2 score	1.3 ± 1.0	1.3 ± 1.0	1.4 ± 1.1	1.2 ± 1.0	1.4 ± 1.0	0.615
Laboratory data						
Hemoglobin, g/dL	14.1 ± 1.7	14.1 ± 1.7	14.2 ± 1.6	14.6 ± 1.7	14 ± 1.4	0.129
Creatinine, mg/dL	0.96 ± 0.78	0.96 ± 0.78	0.89 ± 0.2	0.86 ± 0.2	0.87 ± 0.2	0.289
eGFR, mL/min/1.73 m^2^	65.2 ± 18	65.2 ± 18	65.1 ± 16	66.5 ± 14.8	62.2 ± 13.6	0.52
Plasma BNP, pg/mL	105.1 ± 108.7	105.1 ± 108.7	114.1 ± 130.6	145 ± 148.7	117.1 ± 141.7	0.859
Echocardiography						
LAD, mm	38.8 ± 5.8	38.8 ± 5.8	38.8 ± 6.5	38.6 ± 6.4	37.7 ± 5.7	0.768
LVDd, mm	45.5 ± 5.5	45.5 ± 5.5	45.5 ± 5.6	45.6 ± 5.8	44.7 ± 5.4	0.247
LVEF, %	61.5 ± 9	61.5 ± 9	63.1 ± 9.9	61.3 ± 9.9	63.9 ± 7.8	0.156

Data are presented as n (%), or mean ± SD. BMI = body mass index.

eGFR = estimated glomerular filtration rate, BNP = b-type natriuretic peptide, LAD = left atrial diameter.

LVDd = left ventricular diameter at end diastole, LVEF = left ventricular ejection fraction.

**Table 2:** Pharmacological intervention post-blanking period relative to blood types.

Blood type	All	A	O	B	AB	P value
N, (%)	601 (100 %)	239 (39.8 %)	190 (31.6 %)	122 (20.3 %)	50 (8.3 %)	
Pharmacological therapy						
Beta-blockers, n (%)	243 (40.4 %)	98 (41 %)	74 (38.9 %)	51 (41.8 %)	20 (40 %)	0.966
Anti-arrhythmia drugs, n (%)	96 (16 %)	39 (16.7 %)	27 (14.2 %)	23 (18.9 %)	7 (14 %)	0.709
Ia, n (%)	35 (5.8 %)	19 (7.9 %)	7 (3.7 %)	8 (6.6 %)	1 (2 %)	
Ic, n (%)	7 (1.2 %)	2 (0.8 %)	3 (1.6 %)	2 (1.6 %)	0 (0 %)	
III, n (%)	3 (0.5 %)	2 (0.8 %)	0 (0 %)	0 (0 %)	1 (2 %)	
IV, n (%)	69 (11.5 %)	27 (11.3 %)	22 (11.6 %)	15 (12.3 %)	5 (10 %)	

Data are presented as n (%), or mean ± SD.

### 3.2 Outcomes

**Fig. 2:**
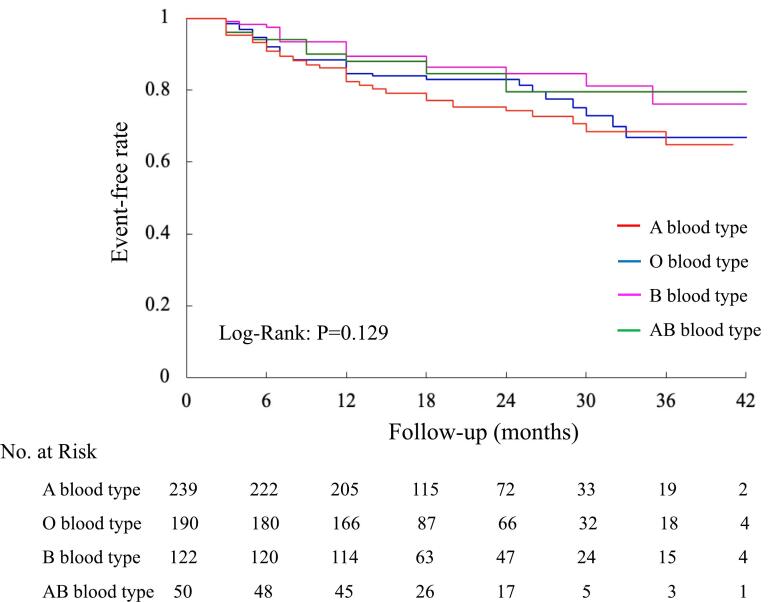
Long-term cumulative incidence of recurrence of atrial fibrillation between the each of blood types. The outcome was established with the use of the Kaplan-Meier curve.

The primary outcome events occurred in 58 (24.3 %), 38 (20 %), 18 (14.8 %), and 8 (16 %) patients with A, O, B, and AB blood types, respectively (hazard ratio [HR], 1.25; confidential interval [CI], 1.02 to 1.52; *P* = 0.03) (Table 3). Kaplan-Meier curve plots depicting the various blood types are illustrated in Fig. 2. This outcome showed that individuals with type B and AB blood types, characterized by the presence of the B antigen, exhibited a lower recurrence of AF compared to those with type A and O blood types, devoid of the B antigen. Subsequently, in accordance with these findings, the subjects were stratified into two groups based on the absence or presence of the B antigen. Table 4 provides an overview of the demographics and baseline data between the non-B antigen blood type (A and O) and B antigen blood type (B and AB) groups. These groups comprised 429 patients (71.4 %) and 172 patients (28.6 %), respectively, and had about 70 % with paroxysmal atrial fibrillation (PAF). No significant differences were discerned in baseline characteristics between absence and presence B antigen blood type groups.

**Table 3:** Summary of primary and secondary outcomes.

Variables	No. (%) of patients					
	A blood type (*n* = 239)	O blood type (*n* = 190)	B blood type (*n* = 122)	AB blood type (*n* = 50)	HR (95 % CI)	P Value
Primary outcome						
AF recurrence, n (%)	58 (24.3 %)	38 (20 %)	18 (14.8 %)	8 (16 %)	1.25 (1.02–1.52)	0.03
	Non-B antigen group (*n* = 429)		B antigen group (*n* = 172)			
Secondary outcome						
AF recurrence, n (%)	96 (22.4 %)		26 (15.1 %)		1.57 (1.02–2.44)	0.021

Data are presented as n (%), or mean ± SD.

HR = hazard ratio, CI = confidence interval, AF = atrial fibrillation.

**Table 4:** Baseline characteristics and pharmacological intervention post-blanking period between the groups.

	Non-B antigen group (A and O)	B antigen group (B and AB)	P value
N, (%)	429 (71.4 %)	172 (28.6 %)	
Variable			
Age, years	66.8 ± 10.7	66.3 ± 11.3	0.625
Male, n (%)	296 (69 %)	117 (68 %)	0.816
BMI, kg/m^2^	24.3 ± 3.9	24 ± 3.5	0.316
Medical history			
Hypertension, n (%)	258 (60.2 %)	99 (57.6 %)	0.655
Diabetes mellitus, n (%)	90 (21 %)	35 (20.3 %)	0.911
Heart failure etiology, n (%)	43 (10 %)	20 (11.6 %)	0.534
Paroxysmal atrial fibrillation, n (%)	303 (70.6 %)	124 (72.1 %)	0.721
CHADS2 score	1.3 ± 1.0	1.3 ± 1.1	0.859
Laboratory data			
Hemoglobin, g/dL	14.1 ± 1.7	14.4 ± 1.6	0.387
Creatinine, mg/dL	0.93 ± 0.6	0.86 ± 0.2	0.187
eGFR, mL/min/1.73 m^2^	65.2 ± 17.2	65.3 ± 14.3	0.958
Plasma BNP, pg/mL	108.8 ± 117.8	128.9 ± 133.2	0.164
Echocardiography			
LAD, mm	38.8 ± 6.1	38.4 ± 6.2	0.549
LVDd, mm	45.5 ± 5.5	44.8 ± 5.8	0.147
LVEF, %	62.2 ± 9.4	62 ± 9.4	0.855
Pharmacological intervention post-blanking period			
Beta-blockers, n (%)	172 (40.1 %)	71 (41.3 %)	0.753
Anti-arrhythmia drugs, n (%)	71 (16.6 %)	30 (17.4 %)	0.773
Ia, n (%)	22 (5.1 %)	9 (5.2 %)	
Ic, n (%)	5 (1.2 %)	2 (1.2 %)	
III, n (%)	2 (0.5 %)	1 (1.6 %)	
IV, n (%)	43 (10 %)	20 (11.6 %)	

Data are presented as n (%), or mean ± SD. BMI = body mass index.

eGFR = estimated glomerular filtration rate, BNP = b-type natriuretic peptide, LAD = left atrial diameter.

LVDd = left ventricular diameter at end diastole, LVEF = left ventricular ejection fraction.

**Fig. 3:**
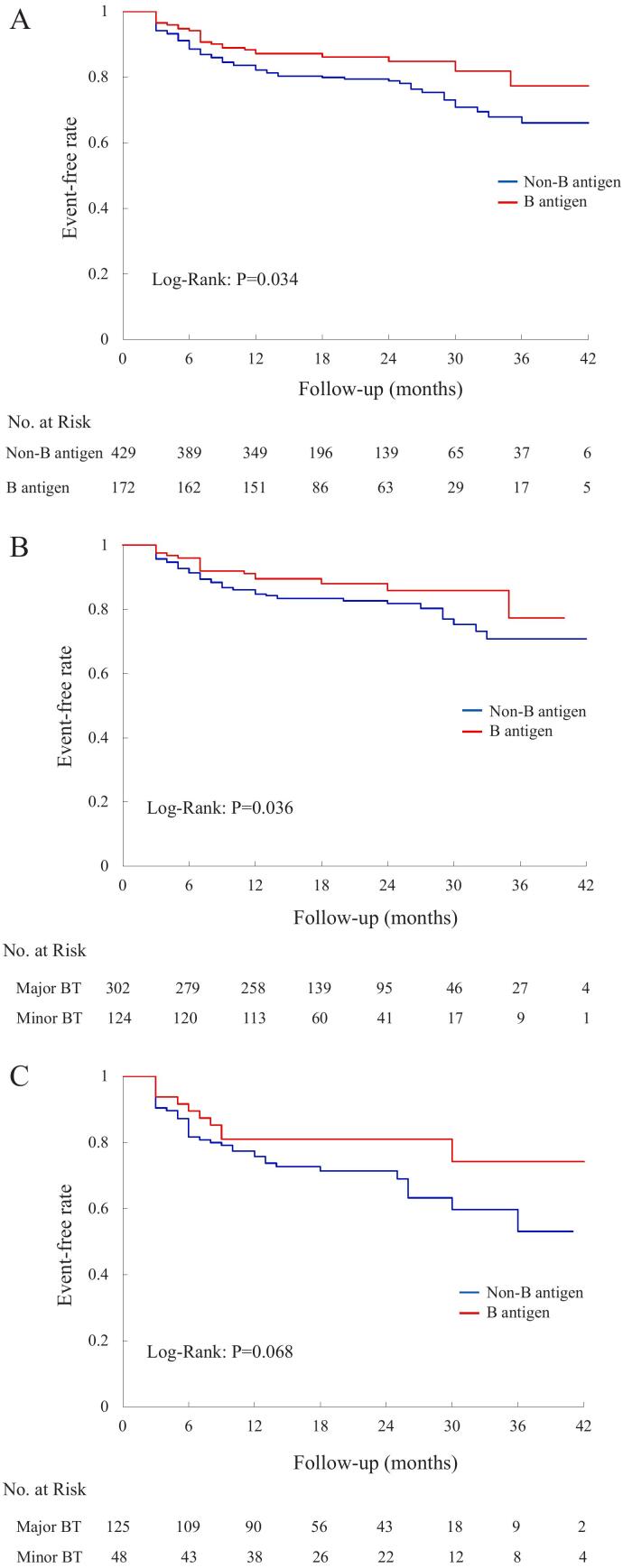
Long-term cumulative incidence of recurrence of atrial fibrillation between the absence and presence of B antigen blood type. Panels A, B, and C illustrate the outcomes across the entire patient cohort, paroxysmal atrial fibrillation (PAF) patients, and non-PAF patients, respectively. All outcomes were established with the use of the Kaplan-Meier curve. PAF indicates paroxysmal atrial fibrillation; and BT, blood type.

The secondary outcome events occurred in 96 patients (22.4 %) in the non-B antigen blood types and 26 (15.1 %) patients in the B antigen blood types (HR, 1.57; confidential interval CI, 1.02 to 2.44; *P* = 0.021) (Table 3 and Fig. 3A). In comparison with the possessing B antigen group, the lacking B antigen group demonstrated a significantly higher rate of AF recurrence (*P* = 0.034) (Fig. 3B, C). Notably, among patients with PAF, the recurrence rate of AF was significantly elevated in the major group (*P* = 0.036), and this trend persisted in non-PAF patients (*P* = 0.068). Univariate and multivariate analyses revealed that lacking B antigen blood types (A and O) (HR, 1.55; 95 % CI, 1.01 to 2.42; *P* = 0.037), hypertension (HR, 1.51; 95 % CI, 1.05 to 2.17; *P* = 0.026) and non-PAF (HR, 1.7; 95 % CI, 1.17 to 2.47; *P* = 0.005) were independently associated with the primary outcome (Table 5).

**Table 5:** Hazard ratio for AF recurrence of catheter ablation.

	Univariate		Multivariate	
	HR (95%CI)	P value	HR (95%CI)	P value
Age > 75 years	1.09 (0.83–1.92)	0.273		
Male	1.17 (0.79–1.72)	0.439		
Non-B antigen blood type (A and O)	1.55 (1.01–2.4)	0.046	1.55 (1.01–2.42)	0.037
BMI > 25	1.13 (0.78–1.63)	0.516		
Hypertension	1.39 (0.95–2.03)	0.095	1.51 (1.05–2.17)	0.026
Heart failure etiology	1.11 (0.61–2.02)	0.734		
eGFR <60 mL/min/1.73 m^−2^	1.39 (0.94–2.05)	0.098		
LVEF <50 %	1.41 (0.78–2.56)	0.26		
LAD >40 mm	1.23 (0.85–1.77)	0.266		
Non-PAF	1.73 (1.21–2.49)	0.003	1.7 (1.17–2.47)	0.005

HR = hazard ratio, CI = confidential interval.

BMI = body mass index, eGFR = estimated glomerular filtration rate, LVEF = left ventricular ejection fraction.

LAD = left atrial diameter, PAF = paroxysmal atrial fibrillation.

## 4 Discussion

The present study delved into the influence of ABO blood type on the risk of atrial fibrillation recurrence following catheter ablation. The key findings were as follows: (1) The incidence of atrial fibrillation associated with blood type remained nearly consistent across the Japanese population. (2) Non-B antigen blood types (A and O) emerged as independent risk factors for the recurrence of atrial fibrillation after catheter ablation.

To the best of our knowledge, this is the first study to have evaluated the relationship between each blood type and recurrence of atrial fibrillation. The recurrence of atrial fibrillation implicates both patient-specific factors and technical aspects of catheter ablation [1,2]. No discernible distinctions were evident in baseline data pertaining to patient-related factors across the study cohorts. The utilization frequencies of beta-blockers and antiarrhythmic drugs post-ablation exhibited comparable patterns across the groups. Technical variables could conceivably have influenced the recurrence of atrial fibrillation. Nevertheless, this study leveraged multi-center data, thereby potentially alleviating the influence and bias linked to catheter ablation technology and individual operators.

The outcomes of this study, spanning an average of 18-month follow-up duration, elucidated a hazard ratio of 1.57 for recurrence in types A and O, devoid of the B antigen, in comparison to types B and AB, characterized by the presence of the B antigen. Disparities in antigenic profiles were postulated as the underlying cause for variations in recurrence rates among different blood types. Distinct sugar chains characterize each blood type [14]. Type A blood possesses *N*-acetylglucosaminyltransferase, responsible for A antigen production, while type B blood possesses galactosyltransferase, the generator of the B antigen. Type AB encompasses both, while type O features neither. These sugar chains are recognized for their involvement in inflammatory processes and tissue healing. Preceding investigations have demonstrated that individuals of non-O blood types exhibit heightened von Willebrand factor activity relative to their O-type counterparts, potentially contributing to enhanced tissue inflammatory responses. Significantly, non-O blood group antigens find expression on the oligosaccharide side chains of von Willebrand factor molecules, leading to a delayed clearance of von Willebrand factor levels [15]. The augmented expression of von Willebrand factor in the atrial intima contributes to the advancement of atrial structural remodeling [16,17]. Atrial fibrillation results in hypercoagulability, and in addition, non-O blood group antigens may enhance von Willebrand factor expression, leading to inflammatory changes and structural remodeling of atrial muscle.

Thrombin, a pivotal factor in this scenario, has been demonstrated to elicit fibrotic and inflammatory responses in adult atrial fibroblasts. Transgenic mouse models have further elucidated that elevated thrombin levels are associated with increased episodes of atrial fibrillation. Conversely, in goats afflicted with atrial fibrillation, reduced thrombin generation resulted in diminished atrial fibrillation complexity and a mitigated presence of atrial fibrillation-related fibrosis [18]. Although these results are based on animal experimentation and cannot be directly applied to humans, these findings underscore the potential role of activated coagulation in the process of atrial remodeling. While catheter ablation is anticipated to induce heightened coagulation in the atrial muscle following radiofrequency ablation, it is noteworthy that all patients received anticoagulation therapy during the blanking period, potentially impeding the progression of inflammation.

All participants in this study underwent pulmonary vein isolation utilizing radiofrequency. Atrial fibrillation observed within a 3-month postoperative blank period has conventionally been regarded as transient, with early recurrence ascribed to edema and inflammation induced by radiofrequency catheter ablation. Nevertheless, recent reports have delineated early recurrence as an important predictor of late recurrences [19,20]. In this study, early recurrence across different blood types was noted in 39 (16.3 %) patients with type A, 23 (12.1 %) with type O, 15 (12.3 %) with type B, and 3 (6 %) with type AB, respectively. Among these individuals, the percentage of late recurrence was 19 (48.7 %) for type A, 9 (39.1 %) for type O, 3 (20 %) for type B, and 1 (25.3 %) for type AB, respectively. These findings unveiled that types A and O exhibited a higher incidence of both early and late recurrence compared to types B and AB. It is postulated that the difference of individual sugar chains may impact the healing process subsequent to radiofrequency ablation. There exist two hypotheses to explain this outcome: first, that the healing process possibly advanced more promptly, resulting in re-connection in type O, lacking antigens, and type A, possessing solely the A antigen; second, that a heightened induction of inflammation in type B and AB, featuring the B antigen, may have rendered re-connection less probable. Anticipating advancements in further research on blood types among patients undergoing redo catheter ablation in the future.

### 4.1 Limitations

The present study had several limitations. Firstly, the skewed distribution of blood types, and the limited representation of AB type, might have impacted the study's outcomes due to the relatively small sample size. Moreover, the variation in blood type distribution across countries adds complexity. Whether analogous results could be replicated in different nations remains uncertain, necessitating further extensive investigations. Secondly, this study did not evaluate the presence or absence of atrial fibrillation symptoms, a crucial factor that could have influenced the disparity in the recurrence rates of atrial fibrillation. Finally, delving into deeper basic research regarding the impact of inflammation on atrial muscle, prompted by the sugar chains of the A and B antigens, would contribute to a more comprehensive comprehension of the underlying mechanisms.

## 5 Conclusions

This study clarifies that, notwithstanding the absence of a correlation between blood types and the incidence of atrial fibrillation, blood types lacking the B antigen manifest an augmented propensity for atrial fibrillation recurrence. Nevertheless, the underlying mechanism linking blood type and recurrence remains elusive, and further comprehensive basial research on blood types is expected.

## Ethical statement

The institutional Ethics Board of Hiroshima General Hospital approved the study protocol and informed consent was obtained from all the patients before enrolment.

## CRediT authorship contribution statement

**Michitaka Amioka:** Writing – review & editing, Writing – original draft, Visualization, Validation, Project administration, Methodology, Investigation, Formal analysis, Data curation, Conceptualization. **Hiroki Kinoshita:** Data curation. **Akinori Sairaku:** Supervision. **Tomoki Shokawa:** Supervision, Data curation. **Yukiko Nakano:** Supervision.

## Declaration of competing interest

The authors declare that they have no known competing financial interests or personal relationships that could have appeared to influence the work reported in this paper.

## Data Availability

No data were generated or analyzed for or in support of this paper.

## References

[1] Balk E.M., Garlitski A.C., Alsheikh-Ali A.A. (2010). Predictors of atrial fibrillation recurrence after radiofrequency catheter ablation: a systematic review. J. Cardiovasc. Electrophysiol..

[2] Calkins H., Hindricks G., Cappato R. (2017). HRS/EHRA/ECAS/APHRS/SOLAECE expert consensus statement on catheter and surgical ablation of atrial fibrillation. Heart Rhythm..

[3] Nery P.B., Belliveau D., Nair G.M. (2016). Relationship between pulmonary vein reconnection and atrial fibrillation recurrence: a systematic review and meta-analysis. JACC Clin. Electrophysiol..

[4] Verheule S., Sato T., Tt Everett (2004). Increased vulnerability to atrial fibrillation in transgenic mice with selective atrial fibrosis caused by overexpression of TGF-β1. Circ. Res..

[5] Liew R., Khairunnisa K., Gu Y. (2013). Role of tumor necrosis factor-α in the pathogenesis of atrial fibrosis and development of an arrhythmogenic substrate. Circ. J..

[6] Kang Q., Li X., Yang M. (2018). Galectin-3 in patients with coronary heart disease and atrial fibrillation. Clin. Chim. Acta.

[7] Fashanu O.E., Norby F.L., Aguilar D. (2017). Galectin-3 and incidence of atrial fibrillation: the Atherosclerosis risk in communities (ARIC) study. Am. Heart J..

[8] Ryu K., Li L., Khrestian C.M. (2007). Effects of sterile pericarditis on connexins 40 and 43 in the atria: correlation with abnormal conduction and atrial arrhythmias. Am. J. Physiol. Heart Circ. Physiol..

[9] Shang L.L., Sanyal S., Pfahnl A.E. (2008). NF-κB-dependent transcriptional regulation of the cardiac scn5a sodium channel by angiotensin II. Am. J. Phys. Cell Phys..

[10] Hu Y.F., Chen Y.J., Lin Y.J. (2015). Inflammation and the pathogenesis of atrial fibrillation. Nat. Rev. Cardiol..

[11] Liu S.H., Lin Y.J., Lee P.T. (2021). The isthmus characteristics of scar-related macroreentrant atrial tachycardia in patients with and without cardiac surgery. J. Cardiovasc. Electrophysiol..

[12] Blais C., Germain M., Delage G. (2016). The association between blood group and the risk of vascular disease in Quebec blood donors. Blood Transfus..

[13] Liu S.H., Chhay C., Hu Y.F. (2023). ABO blood groups as a disease marker to predict atrial fibrillation recurrence after catheter ablation. J. Pers. Med..

[14] Yamamoto F., Clausen H., White T. (1990). Molecular genetic basis of the histo-blood group ABO system. Nature.

[15] Albánez S., Ogiwara K., Michels A. (2016). Aging and ABO blood type influence von Willebrand factor and factor VIII levels through interrelated mechanisms. J. Thromb. Haemost..

[16] Kumagai K., Fukuchi M., Ohta J. (2004). Expression of the von Willebrand factor in atrial endocardium is increased in atrial fibrillation depending on the extent of structural remodeling. Circ. J..

[17] An K., Yin H., Mei J. (2018). Atrial endocardial expression of von Willebrand factor and thrombomodulin is associated with recurrence after minimally invasive surgical atrial fibrillation ablation. Interact. Cardiovasc. Thorac. Surg..

[18] Mujović N., Marinković M., Marković N. (2018). The relationship of early recurrence of atrial fibrillation and the 3-month integrity of the ablation lesion set. Sci. Rep..

[19] Kim Y.G., Boo K.Y., Choi J.I. (2021). Early recurrence is reliable predictor of late recurrence after radiofrequency catheter ablation of atrial fibrillation. JACC Clin. Electrophysiol..

[20] Spronk H.M., De Jong A.M., Verheule S. (2017). Hypercoagulability causes atrial fibrosis and promotes atrial fibrillation. Eur. Heart J..

